# Mitophagy and Traumatic Brain Injury: Regulatory Mechanisms and Therapeutic Potentials

**DOI:** 10.1155/2023/1649842

**Published:** 2023-02-17

**Authors:** Yi Luan, Lulu Jiang, Ying Luan, Yi Xie, Yang Yang, Kai-Di Ren

**Affiliations:** ^1^Clinical Systems Biology Research Laboratories, Translational Medicine Center, The First Affiliated Hospital of Zhengzhou University, Zhengzhou 450052, China; ^2^Department of Anesthesiology and Perioperative Medicine, Henan Provincial People's Hospital, People's Hospital of Zhengzhou University, Zhengzhou 463599, China; ^3^Department of Physiology and Neurobiology, School of Basic Medical Sciences, Zhengzhou University, Zhengzhou 450001, China; ^4^Department of Neurology, The First Affiliated Hospital of Zhengzhou University, Zhengzhou 450052, China; ^5^Department of Pharmacy, The First Affiliated Hospital of Zhengzhou University, Zhengzhou 450052, China; ^6^Henan Key Laboratory of Precision Clinical Pharmacy, Zhengzhou University, Zhengzhou 450052, China

## Abstract

Traumatic brain injury (TBI), a kind of external trauma-induced brain function alteration, has posed a financial burden on the public health system. TBI pathogenesis involves a complicated set of events, including primary and secondary injuries that can cause mitochondrial damage. Mitophagy, a process in which defective mitochondria are specifically degraded, segregates and degrades defective mitochondria allowing a healthier mitochondrial network. Mitophagy ensures that mitochondria remain healthy during TBI, determining whether neurons live or die. Mitophagy acts as a critical regulator in maintaining neuronal survival and healthy. This review will discuss the TBI pathophysiology and the consequences of the damage it causes to mitochondria. This review article will explore the mitophagy process, its key factors, and pathways and reveal the role of mitophagy in TBI. Mitophagy will be further recognized as a therapeutic approach in TBI. This review will offer new insights into mitophagy's role in TBI progression.

## 1. Introduction

Traumatic brain injury (TBI) is one of the leading causes of death and disability in people under 45 years of age. TBI can be induced by external trauma such as falls and motor vehicle accidents [1]. TBI affects about 5.48 million people each year (73 cases per 100,000 people) [2]. Additionally, TBI is responsible for 30.5% of all injury-related deaths [2]. TBI is responsible for a heavy financial burden on the public healthcare system and the economy at large. TBI severity can be measured by the Glasgow Coma Scale (GCS), a commonly used scoring system [3]. Mild TBI or concussions account for approximately 75% of all TBIs. Even mild concussions are highly likely to cause work disability or neuropsychiatric complications. One year after sustaining a TBI, 43% still have cognitive, social, or physical deficits that require further treatment [4].

**Figure 1:**
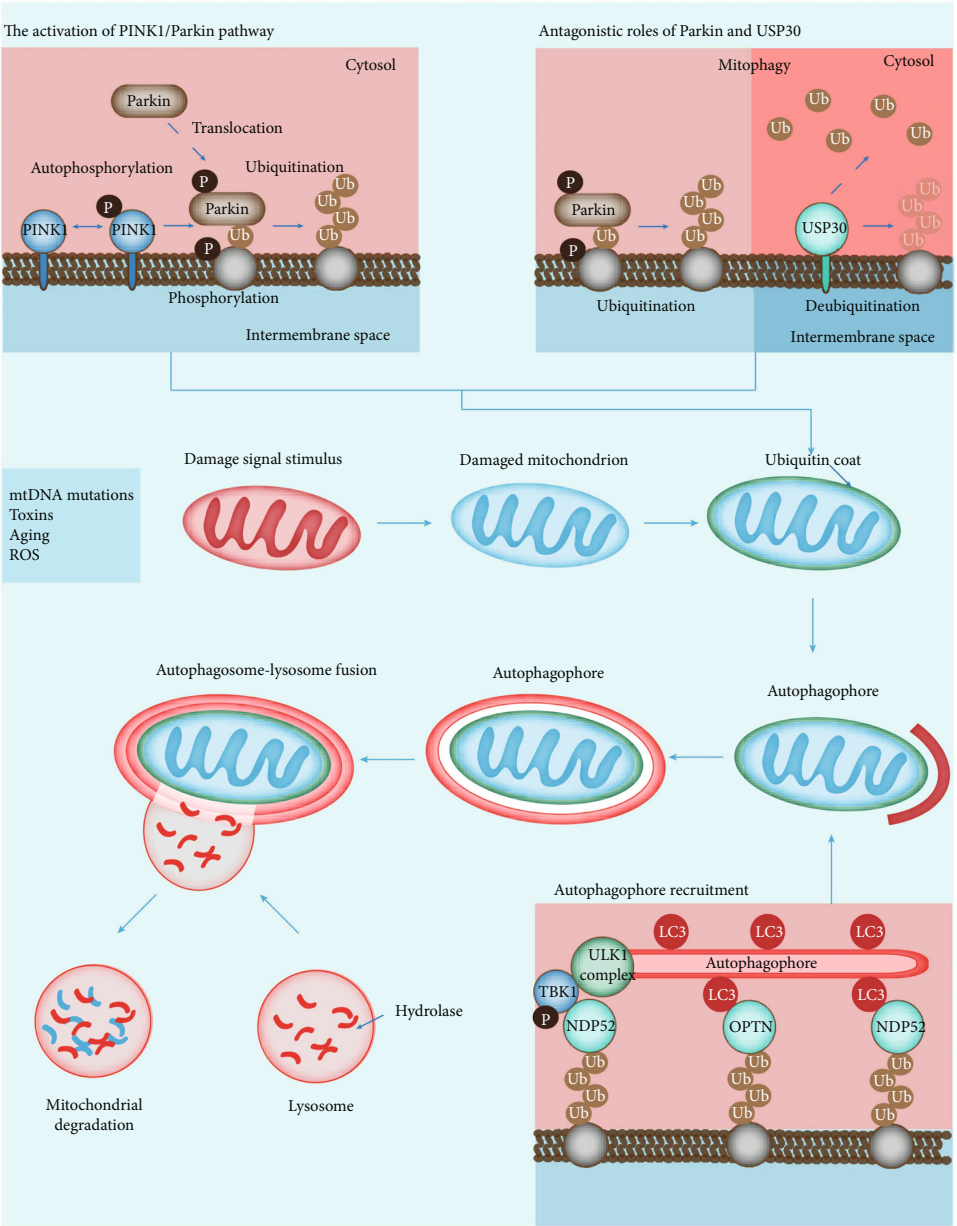
Graphic illustration of pathophysiology of traumatic brain injury (TBI). When mitochondria are stimulated by different stimuli, such as mtDNA mutation, toxins, and reactive oxygen species (ROS), PINK1 is anchored on the outer mitochondrial membrane and autophosphorylated. Moreover, PINK1 contributes to the phosphorylation of Ub and formation of polyubiquitin chains in the whole cell. Simultaneously, the binding of phosphorylated Ub and Parkin promotes its transfer to the mitochondria surface. As Parkin is recruited, the ubiquitination process spreads to the mitochondrial matrix. USP 30 was found to oppose PINK1-Parkin mitophagy. Mitochondrocytes are labeled with ubiquitin recruit mitophagy receptors, such as sequestosome-1 (p62), nuclear domain 10 protein 52 (NDP52), NBR1, and Optineurin (OPTN). These receptors interact with the light chain 3 (LC3) autophagy protein on the outer membrane with their LIR motifs, bind to the polyubiquitin chain through the ubiquitin-binding domain, and initiate mitophagy.

A comprehensive understanding of TBI epidemiology is essential to raise public health policies and prevention strategies. TBI pathogenesis is caused by a combination of primary and secondary injuries that trigger transient or permanent neurological impairments [5]. Among them, the primary deficit refers to direct injury to the brain caused by external stimuli. The second deficit results from molecular, chemical, or inflammatory pathways, which can induce further cerebral damage within minutes or several days after the primary trauma (Figure 1) [6]. This cascade begins with depolarization of neurons, which triggers the release of excitatory neurotransmitters such as glutamate and aspartate, resulting in an increased intracellular calcium concentration [7]. The intracellular calcium triggers a series of mechanisms, including caspases, calpases, and free radicals, ultimately causing apoptosis or direct death of cells (Figure 1). As the disease progresses, the degeneration of cells during TBI causes an inflammatory response that causes further damage to neurons and the brain-blood barrier (BBB) and further cerebral edema [8].

TBI occurs after external mechanical damage to the brain due to anatomical and functional impairment. An injury induced by a TBI is a combination of structural, cellular, and vascular damage [9]. A therapeutic approach focuses on minimizing the primary injury and inhibiting the consequent molecular and cellular cascade of sustained cell damage from alleviating the consequences of a TBI [10]. There is currently no effective intervention for the primary damage. Numerous attempts have been made to protect neurons from post-TBI and maintain previous neuronal reorganization and function.

Several studies have suggested that TBI could cause mitochondrial damage, inflammation, functional cell damage, neurodegeneration, and unfavorable outcomes [11]. In TBI, mitochondrial dysfunction contributes to a series of mitochondrial damage, energy deficits, and apoptosis [12]. Brain has extremely high energy demands, and the energy supply is highly dependent on the efficient utilization of glucose and the integrity of the mitochondria. Maintenance of mitochondrial activity and function remains essential to maintain nerve homeostasis [13]. Mitochondria are highly dynamic organelles that constant proceed fusion and fission during the whole cellular processes [14]. The imbalance of fusion and fission might result in altered mitochondrial morphology and energy deficits and is also implicated in neurodegenerative diseases. Mdivi-1 is shown to inhibit mitochondrial fission in yeast and animal models. Also, Mdivi-1 is reported to reduce cortical cell lose and alleviate spatial memory after TBI in mice.

Several mechanisms contribute to the normal functioning of the mitochondria [15–17]. Under oxidative conditions, the correct folding of mitochondrial proteins is controlled by mitochondrial chaperones and proteases [18]. If these proteins are suppressed, cells trigger unfolded protein response which sustains homeostasis by enhancing ER chaperones to restore the incorrectly folded proteins. Incorporating these processes allows flexibility to adapt to the changing metabolic environment. If these processes are counteracted, mitochondria would be damaged by various stimuli [19]. ROS are produced in excess in damaged mitochondria, ATP levels are reduced, and other metabolic reactions are altered [19].

Autophagy is an important regulator in TBI. In autophagy, membrane-bound autophagosomes can be formed to degrade intracellular proteins and organelles [20]. In clinical and preclinical research, autophagy is increasingly believed to be involved in the pathophysiology of TBI [21]. As detected in a mouse model with a closed head injury, rapamycin therapy enhanced autophagy in TBI mice. Autophagy activation in another study also protected neurons from degradation in the early stages of TBI and continuously eliminated dysfunctional cellular components in subsequent stages [22]. Autophagy suppression prevented neurological damage and cell death caused by weight loss [23]. Therefore, the exact role of autophagy in TBI requires further research.

**Figure 2:**
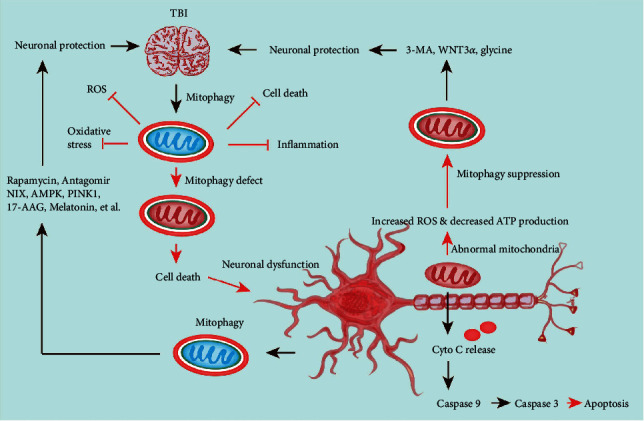
Process of mitophagy. TBI induces the activation of mitophagy in brain tissue. Brain injury contributes to mitochondrial damages and mitochondrial dysfunctions, leading to insufficient mitophagy. As mitochondria are impaired, their energy production is insufficient, causing cell death. Mitophagy can be inhibited using 3-methyladenine (3-MA), WNT3*α*, and glycine is advantageous for neuronal protection. Conversely, rapamycin, antagomir, NIX, AMPK, PINK1, 17-AAG, and melatonin can protect the nervous system by promoting mitophagy.

Mitophagy is a specialized autophagy that maintains normal growth and function by clearing damaged mitochondria [24]. Defects in this process play a vital role in various physiological and pathological processes in the brain. Researchers have also discussed the role of mitophagy in brain trauma [25]. In the case of TBI, mitophagy could prevent neuron injuries and dysfunctions and support mitochondrial functions through obliterating the damaged mitochondria (Figure 2) [26]. Several proteins involved in mitophagy, including Cardiolipin (CL), Parkin, and B-Cell CLL/Lymphoma 2- (BCL2-) interacting protein 3 (BNIP3), and BNIP 3-like (BNIP3L)/NIX, were dysregulated in TBI (Figure 2) [21]. Consequently, mitophagy plays a key role in the pathogenesis of brain trauma. This review is aimed at illustrating the recent advances in mitophagy, including the molecular pathways governing mitophagy and the function of mitophagy concerning TBI and related brain diseases.

## 2. Mitophagy

Mitophagy was first observed in yeast as a selective autophagic process in mitochondrial defects [27]. Mitophagy specifically degrades defective mitochondria and cytotoxic mitochondria to maintain the integrity and homeostasis of mitochondria. Mitophagy defects have been involved in the occurrence of many diseases, such as cardiovascular diseases and neurological disorders [28]. The predominant characteristic of mitophagy is the phenomenon of autophagic vacuole with defective mitochondria, also termed a mitophagosome. Based on the induction factors, mitophagy can be classified into three categories: mitophagy caused by nutrient deprivation, damage signals, and micromitophagy associated with small mitochondrial-derived vesicles [29]. There is an intrinsic difference between these types as the former two require mitochondria to fuse with lysosomes to form an autophagosome, while the latter does not fuse to lysosomes.

Mitophagy is closely regulated by several signaling pathways, including PTEN-induced putative kinase 1 (PINK1)/Parkin, mitophagy receptors, and mitophagy adaptors. Mitophagy receptors mainly orientate on the outer mitochondrial membranes (OMM) by the transmembrane domains and fasten autophagosome to the mitochondria with LC3-interacting region (LIR) motif, such as ATG32 in yeast, BNIP3L/NIX, FUN14 domain-containing 1 (FUNDC1), Bcl2-like 13 (BCL2L13), and FKBP prolyl isomerase 8 (FKBP8/FKBP38), in mammalian cells [30–33]. These mitophagy receptors transfer ATG8 family proteins LC3 and its homolog *γ*-aminobutyric acid receptor-associated protein are incorporated into the mitochondrial membrane and promote the initiation of mitophagy in the presence of LIR motif and independent of the ubiquitin pathway [34].

### 2.1. PINK1

Mitophagy can be either dependent or independent of the phosphatidylinositol-3,4,5-triphosphate 3-phosphatase PINK1 [35]. PINK1-dependent mitophagy is the most widely recognized mitophagy. In healthy cells, PINK1 transfers into the mitochondria and suffers the mitochondrial peptidase-mediated degradation to sustain the relatively low level [36]. PINK1 is, under physiological conditions, present in low levels in the OMM. When there is damage to the mitochondria/loss of mitochondrial membrane potential, PINK1 is stabilized in the OMM [37]. Accumulated PINK1 autophosphorylates, followed by ubiquitin phosphorylation, recruiting Parkin to the mitochondrial membrane. Translocation of Parkin, an E3 ligase, induces its activation and ubiquitination of mitochondrial proteins and subsequent autophagy [38]. In a recent study, mitochondria localized ubiquitin carboxyl-terminal hydrolase 30 deubiquitinase opposed PINK1-Parkin mitophagy (Figure 1). Additionally, Parkin induces mitochondrial depolarization independent of PINK1 to facilitate mitophagy [39].

Parkin's ligase activity can be manipulated by several OMM-localizing proteins, containing MFN1/2, voltage-dependent anion channel protein 1 (VDAC1), and mitochondrial Rho guanosine triphosphate hydrolase (MIRO) [40]. Phosphorylation of MFN2 by PINK1 converts it to a mitochondrial Parkin receptor, insulating damaged mitochondria and initiating Parkin-mediated mitophagy to eliminate them [41]. VDAC1 is polyubiquitinated by Parkin, which induces Parkin-mediated mitophagy by transferring p62/Sequestosome1 (SQSTM1) and LC3B to mitochondria [27]. In contrast, VDAC1 also modulates apoptosis by modulating its pore opening and closing. A polyubiquitination defect in VDAC1 results in apoptosis and hinders Parkin-mediated mitophagy [42]. Accordingly, the modulation of Parkin on VDAC1 determines whether damaged mitochondria undergo apoptosis or mitophagy. Parkin also modulates the ubiquitination and degradation of MIRO1/2 [43]. The knockdown of MIRO protein inhibits Parkin recruitment to mitochondria and mitophagic removal of damaged mitochondria [44].

Further studies reveal that MIRO proteins can also act as a calcium-dependent docking site for Parkin on the mitochondria [44]. Upon calcium binding, MIRO protein is ubiquitinated by Parkin and activates the mitophagy machinery [45]. MIRO1, as a key MIRO protein, functions as safety switch for Parkin recruitment to mitochondria upon mitochondrial release from microtubules. Thus, the dynamics of OMM can modulate mitophagy.

### 2.2. Light Chain 3 (LC3)

**Figure 3:**
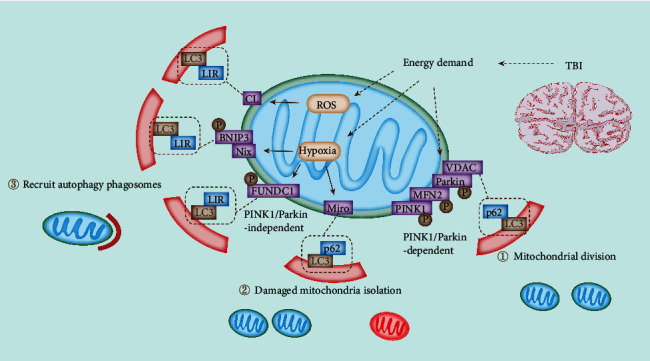
The mitophagy pathways in TBI. Mitophagy is initiated by several pathways, such as ubiquitin-mediated and mitochondrial receptor-mediated pathways. Here are some of the potential mitophagy pathways and complexes involved in TBI. In the aftermath of TBI and due to the energy crisis, the energy demand could not be met, resulting in increased levels of oxidative phosphorylation (OXPHOS) and ROS. Alterations after TBI trigger damage to mitochondria and activate mitophagy pathways.

Ubiquitination remains a critical part in selective autophagy [46]. The recruitment of microtubule-associated protein 1 LC3 (MAP1LC3) by cargo-bound receptors through an LC3-interacting region (LIR) is the most accepted model, linking cargo with a preformed, autophagy-produced membrane [47]. According to this model, receptors are either part of the cargo or are recruited to it by ubiquitination. Furthermore, scaffold proteins play a critical role in the recruitment of additional proteins involved in autophagy. Ubiquitinated proteins are degraded in mitochondria and transported by ubiquitin-binding adaptors through microtubules into the perinuclear zone after OMM remodeling [48]. The binding of adaptor proteins and LC3 promotes the isolation of defective mitochondria in the autophagosomes. Consequently, the fusion of autophagosomes and lysosomes leads to the removal of damaged mitochondria [49]. Cargo-binding receptors (LC3 adapters) are directed to the polyubiquitinated substrates in mitochondria after recognition by LC3 with their ubiquitin-binding domain, such as p62, NDP52, and Optineurin (Figure 3) [50].

### 2.3. Ubiquitin-Independent Mitophagy

In previous studies, PINK1 deficient cells can also activate mitophagy [51]. Defective mitochondria can also be eliminated in an ubiquitin-independent manner by LC3 adaptors [52]. These adaptors directly recognize the damaged mitochondria and recruit the damaged mitochondria to autophagosomes. The clearance of mitochondria by the BNIP3L/NIX pathways is the most extensively researched mitophagy receptors [53]. BNIP3, a mitochondrial BH3-only protein, is relevant to mitochondrial dysfunction and cell death [54]. BINP3 and NIX contribute to mitophagy when stimulated with hypoxia and lead to mitochondrial depolarization and fusion with cell membranes. The interaction between N-terminals of BNIP3 and NIX in the cytoplasm and LC3-related molecules facilitates mitophagy [55]. The direct binding of BNIP3 promotes its stabilization and recruitment of Parkin.

### 2.4. Additional Parkin-Independent Mitophagy

The mitophagy process can also be induced by other Parkin-independent mechanisms [24]. FUNDC1 is an OMM localizing protein that can induce mitophagy through the recruitment of MAP1LC3B/LC3B with its LIR motif in mammalian cells [56]. FUNDC1 activity can be regulated by different kinases. Phosphoglycerate mutase family member 5- (PGAM5-) mediated dephosphorylation is a prerequisite for the activation and interaction with LC3, thus, inducing mitophagy [57]. Choline dehydrogenase (CHDH) is typically in the inner mitochondrial membranes (IMM) and OMM, and CHDH is recruited to OMM when stimulated by mitochondrial degradation [58]. CHDH mediates mitophagy by binding with p62, forming CHDH-p62-LC3 complex [58]. As a nutrient availability sensor, adenosine monophosphate-activated protein kinase (AMPK) phosphorylates TANK-binding kinase 1 (TBK1) and activates it to mediate Parkin-independent mitophagy [59].

In addition to the types mentioned above, there are several LC3-independent forms of mitophagy. To induce mitophagy, for instance, the autophagosome mediated by Rab9 requires the formation of Rab9, Unc-51-like kinase 1 (ULK1), and DRP1 protein complex [60]. Notably, novel mitophagy-associated pathways are being identified. Coiled-coil myosin-like BCL2-interacting protein (BECN1)/Beclin 1 is crucial for the formation and activation of autophagosomes by interacting with Parkin without affecting its recruitment to mitochondria [61]. Emerging evidence has established the important role of ER stress in mitophagy through mitochondrial-associated endoplasmic reticulum (MAM), which control calcium fluxes and apoptosis [15]. The role of MAM in autophagy has been well documented, due to its crucial role in initiating mitophagy [24]. Multiple autophagy-related proteins have been identified in MAMs. PINK1 and Beclin 1 are believed to be located on the mitochondrial cell membranes to facilitate mitochondrial-ER contact and the initiation of mitophagy in response to mitophagy inducers [62].

### 2.5. Mitochondrial Biogenesis

Mitochondrial biogenesis is the process during which new mitochondria are generated. This process can be launched by many signals, such as toxins and mtDNA mutation accumulation [63]. Mitochondrial biogenesis is modulated by four genes, including PPAR-peroxisome proliferator-activated receptor-*γ* coactivator-1*α* (PGC-1*α*), nuclear respiratory factor 1 (NRF1), nuclear respiratory factor 2 (NRF2), and mitochondrial transcriptional factor a (TFAM) [63]. Of these, PGC-1*α* is the most well-documented and widely recognized as the main regulator of mitochondrial biogenesis.

PGC-1*α* acts as a perfect docking platform for the transcription of nuclear encoded genes required for mitochondrial biogenesis. Specifically, PGC-1*α* activation recruits NRFs, which is essential to induce the transcription of several mitochondrial genes, like ATP synthase, cyt c, and TFAM. Then, TFAM moves to the mitochondrial matrix and promotes mtDNA replication and mitochondrial gene expression [63]. PGC-1*α* also combines with other transcriptional factors, such as PPARs, thyroid hormone, and glucocorticoids. The activation of abovementioned genes induces the progression of mitochondrial biogenesis and enhanced mitochondrial functions. Several reports revealed that PGC-1*α* is decreased in both central and peripheral diseases featured by mitochondrial dysfunction.

## 3. The Mitophagy Pathways in TBI

Most importantly, several pathways can induce mitophagy, including ubiquitin-mediated pathways and mitochondrial receptor-mediated pathways (Figure 3) [64]. As a result of TBI, the energy crisis has raised OXPHOS levels, and ROS levels have exceeded a certain threshold. During TBI, cerebral hypoperfusion initiates hypoxia accompanied with decreased mitochondrial membrane potential (ΔΨ*m*) in the brain [65]. Alterations following TBI promote mitochondrial damage and activate mitophagy-related pathways (Figure 3).

Alteration of the OXPHOS-induced energy state activates the Rheb pathway [66]. The reduction in the mitochondrial membrane potential leads to enhanced accumulation of PINK1 kinase on the OMM [67]. Activation of PINK1/Parkin at the OMM facilitates the binding with LC3 for autophagic degradation mediated by specific adaptor proteins, such as p62, which binds both ubiquitin and LC3. The recruitment and activation of Parkin by PINK1 can modulate mitophagy via ubiquitinating Mfn2, Miro1, and VDAC [68]. Stress-induced mitophagy, such as ROS and hypoxia, is mediated by mitochondrial receptors, such as BNIP3, BNIP3L/NIX, FUNDC1, and CL, which can directly interact with LC3 with its conserved LC3 interacting region (LIR) motif [25]. Once the mitophagy pathways mentioned above are activated, the impaired mitochondria can be selectively removed to control the quality of the mitochondria precisely. As a result, the homeostasis within neurons is maintained, which is advantageous for recovering TBI damage.

### 3.1. PINK1/Parkin-Mediated Mitophagy in TBI

In PINK1/Parkin-mediated mitophagy, decreased mitochondrial membrane potential upregulates the expression of PINK1 and recruits it to the OMM, promotes the phosphorylation of Parkin on Ser65, and leads to activation of Parkin by inducing conformational changes [69]. Thus, OMM-localized PINK1/Parkin facilitates the binding with LC3 with specific adapters (Figure 3) [39]. In previous studies, PINK1 and Parkin are induced after TBI, and Mdivi-1 inhibits the activation of these pathways [70]. This suggests TBI stimulation induces the activation of PINK1/Parkin mediated mitophagy. Ji et al. observed that blocking PINK1 and Parkin translocation to mitochondria due to PGAM5 deficiency ameliorated neuroinflammation in TBI mice [71].

The downregulation of PINK1/Parkin mitophagy by RvD1 treatment might alleviate cognitive impairment after TBI by lessening neuroinflammation and preserving astrocytic mitochondria [72]. Treatment with triiodothyronine (T3) slowed the progression of neurological deficits in mice subjected to TBI [73]. However, *PINK1* knockout mice failed to respond to T3, suggesting that T3 inhibits mitochondria-mediated neuronal apoptosis dependent on PINK1-mediated mitophagy [73]. There remains a debate about the precise role of PINK1/Parkin-mediated mitophagy in TBI-induced neurological damage. A reduction in the level of PINK1 and Parkin after drug treatment may result from an improved mitochondrial membrane potential, which is the feedback signal after the mitochondrial functions are restored but are unrelated to improvements in brain functions.

PINK1/Parkin-mediated mitophagy is also altered in many other neurological disorders such as Parkinson's disease (PD) and Alzheimer's disease (AD) [69]. Parkin upregulation is beneficial for restoring mitochondrial dysfunction in AD [74]. Amyotrophic lateral sclerosis (ALS) is distinguished by a more controversial phenotype. Downregulation of PINK1 or Parkin relieved neurodegenerative phenotypes. Activation of Drp1/PINK1/Parkin signaling pathway brought about brain damage in cerebral ischemia/reperfusion injury [75]. Therefore, more studies are recommended to illustrate the precise function of PINK1/Parkin-mediated mitophagy in TBI.

### 3.2. Receptor-Mediated Mitophagy in TBI

Mitophagy can also be modulated by a series of mitochondrial receptors, including BNIP3, BNIP3L/NIX, FUNDC1, nitrophenylphosphatase domain, nonneuronal SNAP25-like protein homolog 1 (NIPSNAP1), NIPSNAP2, BCL2L13, Prohibitin 2 (PHB2), FKBP8, Rheb, and Cardiolipin, which can interact with LC3 with the conserved LIR motif (Figure 3) [76]. BNIP3, BNIP3L/NIX, BCL2L13, and FUNDC1, localized on the OMM, can bind to proteins from Atg8 family on the phagosome through the LIR motif to remove damaged mitochondria by hypoxia [55]. NIPSNAP1 and NIPSNAP2, PHB2, FKBP8, and Cardiolipin are IMM localizing proteins [77].

In general, OMM rupture of OMM is necessary for IMM proteins to recruit the mitophagy molecular machinery of mitophagy [78]. Nevertheless, it may not be essential to rupture the OMM before mitophagy occurs in some circumstances. They may alternatively bind directly to LC3 as autophagy receptors [79]. Relatively few reports have been published on the mitochondrial receptor-mediated mitophagy pathway. NIX expression was reduced by TBI, and NIX overexpression provided neuroprotection in TBI-induced injury by autophagy and apoptosis pathways [80]. A significant increase in NIX expression was observed in intracerebral hemorrhage and ischemia/reperfusion brain injuries, suggesting that overexpression of NIX could serve as a promising therapeutic target in hemorrhagic and ischemic stroke [81]. Numerous brain diseases, such as cerebral ischemia and cerebral hemorrhage, share the exact basic pathological mechanism of hypoxia. However, alterations in NIX levels in various disease models are not the same, suggesting that NIX-mediated mitophagy is associated with hypoxia and other pathological processes, such as cell death pathways.

TBI research focuses on the mitochondrial lipid signaling pathway [82]. As one of the unique lipids in the mitochondrial intima, CL takes up about 15–20% of the lipid in the mitochondrial lipid. Free circulating CL is released into the plasma after TBI, which ruptures the blood-brain barrier [83]. CL was detectable in 18% of severe TBI patients, suggesting the potential role of CL in TBI pathogenesis [84]. Selective CL oxidation initiates three hours after TBI injury in the TBI model. In mammalian cells, externalization of CL to OMM serves as an elimination signal to promote mitophagy by directly interacting with LC3 [85].

Both the human brain and male rats treated with TBI show increased mitophagy [84]. Inhibiting CL synthesis or CL transfer to the OMM significantly suppressed TBI-induced mitophagy. After TBI, suppression of mitochondrial clearance led to worse outcomes, supporting the idea that mitophagy is beneficial. In addition, mutation of LC3 on R10 and R11 impairs the interaction of LC3 peptide with CL *in vitro* and subsequently impairs phagosome recruitment by LC3 [79]. Externalization of CL to OMM induces both mitophagy and *α*-synuclein aggregation and modulates electron transport in PD [86].

Furthermore, Rheb, an OMM and matrix localizing protein, regulates mitophagy according to the state of energy supply and maintains the homeostasis of the mitochondrial energy supply [66]. Rheb is transferred to the damaged mitochondria to remove stress mitochondria within the mitophagosome in the axons of neurons. TBI models have not explored the influence of mitochondrial receptors on mitophagy except for BNIP3L/NIX and CL. Therefore, mitochondrial receptor-mediated mitophagy remains to be explored in TBI.

## 4. Role of Mitophagy in TBI

As discussed previously, TBI originates from primary injuries, most commonly induced by external injuries, and then gradually evolves into secondary damage, accompanied with inflammatory responses, oxidative stress, and cell death [87]. During TBI progression, these biological processes cooperate with mitophagy to maintain proper mitochondrial quality, which determines the fate neurons. TBI damages mitochondrial quality through oxidative stress, inflammatory response, and cell death. Inflammatory response and oxidative stress can be further induced by damaged mitochondria, which release reactive oxygen species and stimulate the production of proinflammatory cytokines. Therefore, mitophagy-accelerating therapies may provide a promising approach to combat neurological dysfunctions caused by TBI as they mitigate downstream oxidative stress, inflammatory responses, and cell death.

### 4.1. Inflammation and TBI

TBI-induced mitochondrial damage has recently become a focus in TBI research, and restoring mitochondrial function offers a novel approach for treating TBI [88–91]. In the past, researches have focused on drugs that inhibit the posttraumatic inflammatory response. Anti-inflammatory drugs also contribute to mitochondrial function through their effects on mitophagy pathways. Resolvin D1, for instance, improves neuroinflammation and facilitates the clearance of damaged mitochondria by activating mitophagy [92]. Pifithrin-*μ* and pifithrin-*α*, activators of IL-10 and p53, both alleviate neurological damage by modulating neuroinflammation and mitophagy [93]. The neuroprotective effects of rapamycin have been ascribed to its inhibition of mitophagy and suppression of NLRP3-mediated inflammation [94]. Furthermore, the inhibited effect of melatonin on inflammation is mediated through the induction of mitophagy to remove damaged mitochondria.

Inflammation may result in cumulative damaged mitochondria, and insufficient mitophagy will stimulate the activation of proinflammatory pathways [95]. Mitophagy inhibition will exacerbate inflammation caused by TBI, indicating a clear correlation between mitophagy and inflammation [96]. PINK1-mediated mitophagy interacts with NLRP3 inflammasome, and mitophagy can inhibit NLRP3 inflammasome and protect the brain after traumatic injury [73]. Melatonin protects against TBI-induced immunopathology by suppressing inflammation through mitophagy [97]. Ding et al. reported that fisetin inhibits inflammation by inducing mitophagy in sepsis-associated encephalopathy [98]. Another example is that FUNDC1 alleviates inflammation after intracerebral hemorrhage (ICH) by inducing mitophagy in mice, indicating FUNDC1 may be a potential target for ICH treatment [99]. Thus, therapies targeting mitophagy might reduce inflammation by preventing downstream cascades triggered by damaged mitochondria.

### 4.2. Oxidative Stress and Mitophagy

In TBI, the increase in energy requirements results in superabundant ROS production, leading to mitochondrial DNA damage and mitochondrial dysfunction [88, 100, 101]. Mitophagy effectively eliminates damaged mitochondria and sustains the normal function pf neurons [102]. Additionally, mitophagy can relieve gastrointestinal dysfunction induced by TBI. Further investigations have demonstrated that mitophagy could alleviate TBI-induced intestinal mucosal injury and epithelial barrier dysfunction via suppressing ROS production. Due to the traumatic circumstances and stimulation factors, the extent of mitophagy activation varies.

Moderate mitophagy can effectively remove the impaired mitochondria and maintain the normal function of neurons [103]. Mitophagy is insufficient to eliminate defective mitochondria, causing accumulated ROS, which will eventually cause mitochondrial DNA to oxidize and mitochondrial damage to accumulate [104]. During excessive mitophagy, excessive mitochondria are removed. Since mitochondrial are essential to sustain the normal function of neurons, excessive clearance will lead to neuronal death and ultimately damage brain function.

Mitochondrial division inhibitor 1 (Mdivi-1) could improve TBI-induced injuries to the BBB and relieve cell death by suppressing mitophagy, as well as mitochondrial division [105]. Another study reported that mitophagy was protective against TBI-induced brain damage, while Mdivi-1 had the opposite effect in TBI rats and aggravated brain damage [106]. In light of this, we speculated that the diverse roles of mitophagy on TBI-induced damage might be related to the extent of mitophagy.

A substantial body of evidence demonstrates that mitophagy provides favorable effects in removing damaged mitochondria and ROS. In TBI models, a proper degree of mitophagy activation or excessive inhibition may benefit the survival of damaged mitochondria and neurons [25, 93, 107, 108]. Mitophagy pathways differ between neurons and immortalized cells in spatial and kinetic ways, and thus, their outcome and function might differ significantly [109]. Mitophagy in neurons usually has a higher threshold compared with other cells and is prone to partial repair and degradation. As reported by a recent study, 5,6-dicarboxy-1,1,3,3-tetraethylisoindolin-2-yloxyl (DCTEIO), a superoxide dismutase stimulant, could remove defective mitochondria, maintain the normal mitochondrial function, and alter tissue damage and nerve function after TBI by clearing accumulated ROS, lowering oxidative stress, and facilitating mitophagy [110]. *In vivo* study further verified the improvement of DCTEIO in mitophagy promotion and damage extent in the brain 24 hours and 6 weeks after DCTEIO treatment shortly after injury. In another study, Lin et al. observed that triiodothyronine (T3) significantly reduced ROS production by enhancing PINK1-mediated mitophagy, indicating that T3 plays a beneficial role in neuronal protection [73].

### 4.3. Cell Death and Mitophagy

Cell death is the final destiny of a neuron when various stress exceeds the cell repair capacity [111]. Traumatic damage induces a sudden reduction in energy production in damaged neurons and neuron death, which may also occur in neurodegenerative diseases. TBI results in two types of neuron death, necrosis, and programmed cell death [112]. Early neuroprotective intervention may reduce the risk of necroptosis caused by irreversible metabolic disturbances and membrane disruptions [113]. In neurons, programmed cell death occurs on a specific time. Recent studies indicate that neuronal cell death occurs during necroptosis and other modes of death, including apoptosis, pyroptosis, ferroptosis, entotic cell death, necrotic cell death parthanatos, lysosome-dependent cell death, autophagic cell death, alkaliptosis, and oxeiptosis [114].

Mitophagy is essential for maintaining the fitness of cells and also important in modulating cell death. Apoptosis, one of programmed cell death (PCD), leads to shrinkage, chromosome condensation, and DNA fragmentation [115]. Mdivi-1, which explicitly suppresses mitophagy, can activate apoptotic markers caspase-3 and caspase-9, indicating that mitophagy significantly reduces TBI-induced cell apoptosis [106]. Mdivi-1 was also found to decrease LC puncta and TUNEL-positive cells, implying that Mdivi-1 inhibition on autophagy may be associated with the antiapoptotic effect in TBI [116]. In addition, the CL-dependent mitophagy in TBI model plays a neuroprotective role that relieves neuronal apoptosis and behavioral deficits [84]. Moreover, treatment with T3 can offer a therapeutic method for inhibiting apoptosis by PINK1-mediated mitophagy [73].

Pyroptosis, another form of programmed cell death, is induced by proinflammatory cytokines and related inflammatory responses [117]. Microglia undergo pyroptosis in response to stimuli and oxygen deprivation and hypercapnia, suggesting that mitophagy negatively regulates pyroptosis. Rapamycin-induced mitophagy promotes neuroprotection and inhibits activation of pyroptosis after TBI [94]. Also, quercetin alleviates neuronal injury through suppressing pyroptosis in microglia and enhancing mitophagy in models of depression and PD [118].

Notably, a newly characterized cell death known as ferroptosis is characterized by iron accumulation and lipid peroxidation within the cell death process [119]. Osteoblastic ferroptosis caused by ferritin deprivation is caused by PINK1/Parkin-mediated mitophagy in type 2 diabetic osteoporosis [120]. The relationship between mitophagy and ferroptosis in the brain has yet to be explored. The association between ferroptosis and multiple diseases may lead to a new approach to drug discovery in ferroptosis.

### 4.4. Other Factors in TBI

Recently, escalating evidences revealed that aging-related factors are involved in defective mitophagy. In addition, defective mitophagy is also implicated in accelerating physiological aging and neurodegenerative diseases [121]. The unique structure of neurons and their high dependance on energy makes mitochondrial critical important for its proper functions, and dysfunction in mitochondria can result in neurodegeneration. TBI may be responsible for launching the pathological cascade, which leads to unhealthy brain aging [122]. Longitudinal prospective studies are needed to provide more in-depth understanding of the relationship between TBI and the progression of neurodegenerative disorders.

## 5. Therapeutic Approaches Targeting Mitophagy in TBI

Targeting pathways that can activate mitophagy to alleviate harmful consequences such as oxidative stress, inflammatory response, and cell death may impair the damage caused by TBI and improve neurological dysfunctions. Therefore, we conclude that mitophagy activators and inhibitors may be helpful for TBI therapy.

**Figure 4:**
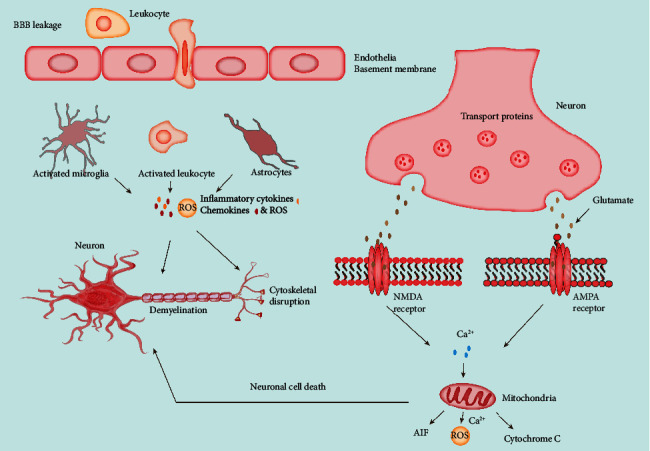
Mitophagy in TBI. Activated leukocytes can translocate to the injured brain parenchyma after a TBI causes BBB dysfunction. Activated leukocytes, microglia, and astrocytes then generate ROS, inflammatory cytokines, and chemokines, impairing neuronal function and further neurodegeneration. In addition, glutamate and aspartate neurotransmitters accumulate in the synaptic space and activate N-methyl-D-aspartate (NMDA) and AMDA receptors on postsynaptic membranes, allowing calcium ions to enter the synaptic space. The production of ROS and the activation of calpains are ultimately triggered by calcium. In response to mitochondrial dysfunction, apoptosis-inducing factor (AIF) and cytochrome c are secreted into the cytosol. These cellular and molecular events ultimately lead to neuronal cell death.

Following traumatic brain injury, Resolvin D1, an activator of mitophagy, mitigated cognitive impairment by protecting astrocyte mitochondria [92]. Transplantation of mesenchymal stem cells overexpressing IL-10 induced autophagy response and reduced neuronal degeneration and death in a rat model of TBI. Another study indicated that pifithrin-*μ* and pifithrin-*α*, inactivators of p53, mitigated TBI-induced neuronal damage by modifying oxidative stress, neuroinflammation, and mitophagy [93]. Rapamycin attenuated neuroinflammation and mitochondrial damage, demonstrated neuroprotective effects, and inhibited the activation of the NLRP3 inflammasome after TBI (Figure 4) [94]. Melatonin repressed inflammation, ameliorated neuronal death and behavioral deficits, dampened the secretion of proinflammatory cytokines, and, therefore, attenuated traumatic brain injury [97]. Furthermore, Mdivi1 alleviated mitochondrial membrane potential loss, ROS production, ATP reduction, BBB disruption, and cell death [116]. In another study, Mdivi1 worsened neurological symptoms and neuronal apoptosis after a traumatic brain injury [123]. Nitroxides scavenged ROS, improved tissue repair, and preserved neurological apoptosis in a rat TBI model [110]. Through mitophagy, T3 ameliorated neurological deficits in mice exposed to TBI [73]. Morin, a natural polyphenol, enhanced autophagy, reduced inflammation and apoptosis, and improved memory after mild traumatic brain injury [21]. In the rat model of controlled cortical impact (CCI), a well-recognized TBI model, post-TBI administration of 17-allylamino-demethoxygeldanamycin (17-AAG) attenuated brain edema and decreased neuronal death, resulting in improved recovery of motor function (Figure 4) [124]. Collectively, these studies have demonstrated therapeutic approaches targeting mitophagy in TBI.

Accumulation of defective mitochondria primarily owning to inadequate mitophagy is associated with TBI. Therefore, mitophagy enhancers, like urolithin, tomatidine, and NAD^+^ riboside can be used to combat TBI. Moreover, several other chemicals targeting mTORC1 (AMP-activated protein), including rapamycin, torin1, perhexiline, niclosamide, and rottlerin, might be helpful in enhancing mitochondrial biogenesis in disease state [125]. For instance, resveratrol and quercetin have brought beneficial effects by activating mitophagy in cardiac and hepatic cells. Further investigations on developing mitophagy enhancers are essential for identification of potential mitophagy enhancers in TBI.

Apart from the above drug-based therapeutic approaches, other approaches are currently under investigation for TBI such as ultrasounds or infrared light that stimulates cytochrome C and overall mitochondrial activity reducing inflammation. The use of a novel, proprietary noninvasive nanopulsed laser therapy (NPLT), which connects near-infrared laser light (808 nm) and laser-generated, low-energy optoacoustic waves, mitigates TBI-induced impairments in neurogenesis and cognitive function in the rat fluid percussion injury model [126]. NPLT turns to be an effective therapeutic approach in alleviating TBI-induced cognitive dysfunction and dysregulation of neurogenesis, and modulation of miRNAs acts as a promising method in neuroprotective effects. Nanopulsed laser therapy is protective in a rat model of blast-induced neurotrauma [127].

## 6. Outlook

After traumatic brain injury (TBI), insufficient oxygen supply to the brain leads to reduced energy production (ATP), leading to nerve cell death. The importance of oxygen delivery after TBI cannot be overstated, and mitochondria play a critical role. Increasing oxygen delivery will not necessarily increase ATP production from damaged mitochondria, as they are the primary sources of ATP in patients. Animal studies have further shown that traumatic brain injury impairs mitochondrial function, and mitochondrial autophagy is essential for maintaining mitochondrial health.

A dysfunctional mitochondrial system will result in a dysfunctional axon and even the whole neuron. The neuron is therefore responsible for strict mitochondrial quality control. Neurons remove damaged mitochondria through lysosomal pathways, namely, mitophagy, one of the main mechanisms for controlling mitochondrial quality. Autophagy in mitochondria is still largely unknown and is one of the hot topics in neuropharmacology and neurobiology. Further research is therefore needed to determine whether mitophagy can specifically improve mitochondrial quality in neurons, reduce cell apoptosis, and ultimately play a neuroprotective role against traumatic brain injury.

Regulation of mitochondria-mediated autophagy may be applicable in the treatment of TBI. However, due to the complexity of mitophagy mechanisms, mitochondrial autophagy is both detrimental and beneficial, and future insights can move to maximizing its benefits. Researches on mitophagy in the nervous system mainly aim to reveal the molecular pathogenesis and potential targets for the therapy of neurological diseases. However, there is still controversy regarding the exact mechanism of targeting mitophagy, differences in research and environmental conditions, and how best to address these issues. In addition, there is a lack of extensive and rigorous research on acute diseases such as TBI. These questions need to be addressed in future studies.

Studies have demonstrated that mitophagy plays a crucial role in nerve cell apoptosis and necrosis after traumatic brain injury and that mitophagy is usually triggered by trauma. Therefore, if mitochondrial autophagy can be used as a novel therapeutic target to increase mitochondrial function, regenerate mitochondria, reduce mitochondrial death, and prevent nerve cell death, it could be a breakthrough in the treatment of patients with brain injuries. However, there is still no direct evidence for the role of mitophagy in TBI. The precise mechanism and regulatory pathway of mitophagy should be further investigated to clarify the role of mitophagy in damaged brain tissue, improve the prognosis of TBI patients, and identify new therapeutic targets.

## Data Availability

All data generated or analyzed in this study are available from the corresponding author on reasonable request.
